# Higher ETCOc predicts longer phototherapy treatment in neonatal hyperbilirubinemia

**DOI:** 10.3389/fped.2023.1154350

**Published:** 2023-04-11

**Authors:** Yuan-Li Zhan, Hai-Bo Peng, Zhen-Chao Jin, Jin-Feng Su, Xiang-Yu Tan, Lu Zhao, Lian Zhang

**Affiliations:** Department of Neonatology, Affiliated Shenzhen Baoan Women's and Children's Hospital, Jinan University, Shenzhen, China

**Keywords:** ETCOc, phototherapy, hyperbilirubinemia, neonates, predict

## Abstract

**Objective:**

This study aimed to evaluate the predictive performance of end-tidal carbon monoxide corrected to ambient carbon monoxide (ETCOc) values phototherapy in neonates with significant hyperbilirubinemia.

**Methods:**

A prospective study was conducted on neonates with significant hyperbilirubinemia who received phototherapy between 3 and 7 days of life. The breath ETCOc and serum total bilirubin of the recruited infants were measured on admission.

**Results:**

The mean ETCOc at admission in 103 neonates with significant hyperbilirubinemia was 1.70 ppm. The neonates were categorized into two groups: phototherapy duration ≤72 h (*n* = 87) and >72 h (*n* = 16) groups. Infants who received phototherapy for >72 h had significantly higher ETCOc (2.45 vs. 1.60, *P* = 0.001). The cutoff value of ETCOc on admission for predicting longer phototherapy duration was 2.4 ppm, with a sensitivity of 62.5% and specificity of 88.5%, yielding a 50% positive predictive value and a 92.7% negative predictive value.

**Conclusion:**

ETCOc on admission can help predict the duration of phototherapy in neonates with hyperbilirubinemia, facilitate clinicians to judge disease severity, and make clinical communication easier and more efficient.

## Introduction

Neonatal hyperbilirubinemia is a common disease with many etiologies that ultimately results in an imbalance between the production and elimination of bilirubin. Even in normal term neonates, bilirubin production is increased compared with adults because of neonates' shortened red blood cell (RBC) lifespan. Red cell breakdown and heme catabolism produce bilirubin and free carbon monoxide (CO) in equimolar ratios (1). Free CO combines with hemoglobin to form carboxyhemoglobin and is later exhaled. The rate of bilirubin production can be assessed quantitatively by measuring blood carboxyhemoglobin or end-tidal carbon monoxide (ETCO) levels. The noninvasive technique of measuring end-tidal breath CO corrected for room air (ETCOc) has been used for several years to estimate the rate of total bilirubin formation (2, 3).

Elevated ETCOc and carboxyhemoglobin levels were previously identified as clinical predictors of iso-immune hemolysis (4–7), such as maternal-fetal blood group incompatibilities of ABO, Rh, or other minor groups, and can alert physicians to other potential pathologies associated with increased bilirubin production, such as glucose-6-phosphate dehydrogenase (G6PD) deficiency (8, 9), structural and functional red blood cell abnormalities, and perinatal sepsis (10–12). ETCOc measurements can be used to identify the presence of hemolysis in infants and the subsequent risk of hyperbilirubinemia. Furthermore, the combination of ETCOc and dynamic serum total bilirubin (STB) measurements may help in the evaluation of bilirubin production and elimination in infants (4). ETCOc measurement is recommended by the 2004 American Academy of Pediatrics guidelines, as it may improve the early recognition of high-risk infants and the management of hyperbilirubinemia in neonates receiving phototherapy (13). Although ETCOc measurement has been shown to improve the efficacy of diagnosing neonatal hemolytic diseases, and it has some predictive value for hyperbilirubinemia, it is unclear whether it is helpful in assessing the effectiveness of phototherapy in neonates with hyperbilirubinemia.

In some medical facilities, phototherapy usually requires hospitalization, which leads to the separation of the mother and baby. To reduce the inconvenience of this separation, both physicians and parents must have a preliminary estimate of the subsequent course, which is greatly beneficial for cases of both hemolytic and non-hemolytic jaundice.

Clinically, neonates often undergo intermittent phototherapy. During early disease stages, a transient decrease in STB after phototherapy does not signify the end of the treatment course in neonates with rapidly increasing bilirubin levels. These patients often require multiple rounds of phototherapy before a steady, significant decrease in STB levels is achieved. Although some predictive tools have been developed for cases of rebound jaundice and are associated with an infant's gestational age (GA), age at phototherapy initiation, and STB level relative to the treatment threshold at phototherapy termination (14, 15), ETCOc has not been analyzed as a parameter.

The aims of this study were to evaluate the performance of ETCOc values in predicting the duration of phototherapy in neonates with significant hyperbilirubinemia,and to investigate the risk factors of prolonged phototherapy.

## Methods

### Study design and populations

This study is written according the “STROBE guidelines" (16). This prospective, single-arm study was conducted in the neonatology department of Shenzhen Baoan Women's and Children's Hospital from January 1, 2021 to May 31, 2021. The study protocol and informed consent forms were approved by the institutional review board of Shenzhen Baoan Women's and Children's Hospital (LISC-2021-03-9-09-KS).

The inclusion criteria were as follows: GA ≥35 weeks and birth weight (BW) ≥ 2000g; postnatal age >3 days and <7 days; significant hyperbilirubinemia (STB level requiring phototherapy for the given age and risk category); and admission to the neonatology department for intensive phototherapy.

Infants who had pulmonary diseases and required respiratory assistance (including the use of ventilators, noninvasive assisted ventilation, or oxygenation *via* hood, tent, or cannula); had severe or life-threatening congenital anomalies; received phototherapy before admission; and needed blood exchange at admission were excluded.

### Clinical information and measurements

Measurements of ETCOc and STB were performed for all recruited populations within 6 h of admission. ETCOc measurements were performed using a CoSense ETCO monitor (Capnia, Inc.). The clinicians who managed the infants were unaware of the values of ETCOc. The STB was checked on admission and before discharge for each infant, and some infants (depending on the clinician's judgment) had their STB levels rechecked multiple times throughout their stay in hospital. STB was measured using the oxidizing method (Maccura, Sichuan, China) on a Hitachi 7,600 autoanalyzer. The clinical variables considered were sex, BW, GA, age at admission, mode of delivery, presence of hemolytic diseases (confirmed by positive direct antiglobulin test, which was performed for all infants born to women who were type O or Rh negative), G6PD deficiency (confirmed through a quantitative test using spectrophotometry), and presence of cephalhematoma.

The study focused on the association between ETCOc values and the following phototherapy-related metrics: (1) duration of phototherapy; (2) age at which phototherapy was initiated; (3) time of phototherapy termination (STB ≤220 µmol/L); and (4) the study cohort was categorized into phototherapy > 72 h and ≤72 h groups. Statistical analysis was performed after grouping and receiver operating characteristic (ROC) curves were plotted.

### Sample size calculation

According to our pilot study, we set a sensitivity of 80%, a specificity of 80%, an alpha error of 0.05, and a tolerance error of 20%.The positive sample size needed is 16.

### Statistical analysis

Categorical variables are expressed as proportions (n/N (%)) and compared using the *χ*2-test. Continuous variables are expressed as means (standard deviations [SD]) or medians (interquartile ranges [IQR]) and compared using a Student's t-test or nonparametric test, as appropriate. Two-sided *P*-values < 0.05 were considered statistically significant. Variables that reached *P* ≤ 0.1 in the difference analyses or clinically significant cases were subjected to a multivariable binary logistic analysis. A multivariable binary logistic regression model was developed using the clinical features of infants who received phototherapy for >72 h. Odds ratios (ORs) and 95% confidence intervals (CIs) were generated from this model, which included hemolytic diseases, male sex, BW, ETCOc, GA, and STB (at admission). ROC and AUC were built to assess the prognostic performance of ETCOc, STB alone, and a combination of both. All statistical analyses were performed using R software 3.0.

## Result

### Patient characteristics

A total of 122 infants with hyperbilirubinemia were eligible and enrolled in this study. Of the excluded infants (*n* = 19), 10 were excluded as their parents' did not provide written consent; 4 were excluded because of ETCOc measurement errors due to high hydrogen interference or ETCOc calculation errors; and 5 were excluded because ETCOc measurements were not available within 6 h of admission because of breath rates of <10 bpm or >50 bpm despite repeated tests (maximum of three attempts).

A total of 103 infants with significant hyperbilirubinemia (STB 327.00 [306.95, 354.90] µmol/L) were recruited between 3 and 7 days after birth (mean 3.65 days),with mean GA and BW of 38.9 ± 1.07 weeks and 3255.73 ± 413.45 g, respectively. The cohort included 68 male and 36 female infants; 84 infants were born vaginally and 19 were born *via* cesarean section. Hemolytic diseases were present in 11 infants, including ABO incompatibility in 1 infant and G6PD deficiency in 10 infants. Cephalohematomas were observed in five infants. Blood tests and the ETCOc measurements were performed within 6 h of admission. The mean duration of phototherapy was 68.50 ± 20.18 h; 16 infants received phototherapy for >72 h, and 1 infant received phototherapy for 144 h.

### Phototherapy duration >72 h and risk factors

A total of 103 infants were categorized according to the duration of phototherapy into the ≤72 h (*n* = 87) group and >72 h (*n* = 16) groups. No differences were observed between the two groups with respect to the GW, delivery mode, admission age, presence of hemolytic diseases (blood type incompatibility and G6PD deficiency) and hemorrhagic diseases, and in laboratory tests including reticulocytes (RET), hematocrit (HCT) and hemoglobin levels. Male infants were more likely to receive longer phototherapy (*P* = 0.024). Infants who received phototherapy for >72 h had significantly higher ETCOc at admission (*P* = 0.001) (Table 1).

**Table 1:** Comparative analysis of factors categorized by phototherapy duration ≤72 h and > 72 h.

	Phototherapy ≤72 h	Phototherapy >72 h	*P*-value	Test
N	87	16		
Male (%)	53 (60.9)	15 (93.8)	0.024	
GA (mean (SD))	38.98 (1.01)	39.03 (1.39)	0.873	
C-section (%)	18 (20.7)	1 (6.2)	0.309	
Admission age (day, median [IQR])	3.00 (3.00, 4.00)	3.00 (3.00, 4.00)	0.155	Nonnorm
RET (%, median [IQR])	2.60 (2.05, 3.20)	2.55 (2.08, 3.30)	0.855	Nonnorm
HCT (mean (SD))	0.50 (0.05)	0.48 (0.05)	0.248	
Hemoglobin (g/L, mean (SD))	174.20 (17.92)	170.56 (17.76)	0.457	
Hemolysis disease(%)	9 (10.3)	2 (12.5)	1.000	
Hemorrhagic (%)	5 (5.7)	0 (0.0)	0.726	
STB at admission (µmol/L, median [IQR])	324.90 (306.45, 353.05)	337.05 (313.05, 360.12)	0.33	Nonnorm
ETCOc (ppm, median [IQR])	1.60 (1.10, 2.00)	2.45 (1.87, 3.12)	0.001	Nonnorm

GA, gestational age; SD, standard deviation; IQR, interquartile range; RET, reticulocytes; HCT, hematocrit; ETCOc, End-tidal carbon monoxide corrected to ambient carbon monoxide.

Multivariate analysis of the risk factors associated with the two outcomes is shown in Table 2. Multivariate analysis revealed that a higher ETCOc was a risk factor for a long phototherapy duration (*p* < 0.001). However, sex, GA, and STB at admission were not significant risk factors for a long phototherapy duration.

**Table 2:** Multivariate analysis of risk factors associated with the phototherapy duration.

Term	Estimate	Std. error	Statistic	OR [95% CI]	*P*-value
Male	1.91	1.11	1.71	6.73 (1.07, 132.10)	0.09
GA	−0.17	0.30	−0.56	0.85 (0.46, 1.54)	0.58
STB at admission	0.00	0.01	−0.14	1.00 (0.98, 1.02)	0.89
ETCOc at admission	1.24	0.41	3.06	3.45 (1.66, 8.40)	0.00
(Intercept)	−4.79	11.76	−0.41	0.01 (0.00, 128,940,590.84)	0.68

GA, gestational age; STB, serum total bilirubin; ETCOc, End-tidal carbon monoxide corrected to ambient carbon monoxide; OR, Odds ratio.

### Predictive value of ETCOc at admission for prolonged phototherapy

**Figure 1:**
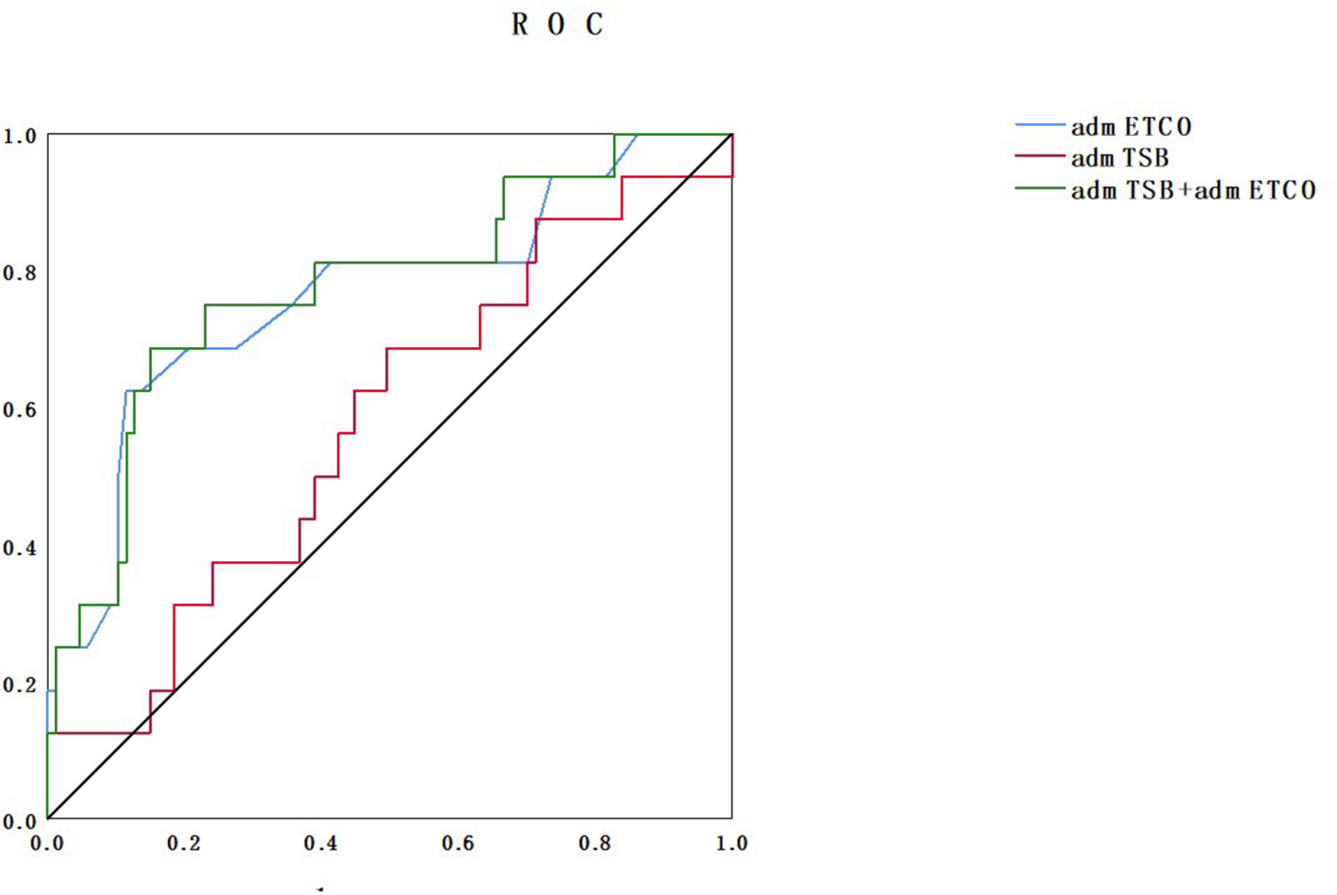
ROC curves comparing ETCOc measurements, STB measurements alone on admission, and combined ETCOc and STB measurements. ROC, receiver operating characteristic; ETCOc, end-tidal carbon monoxide corrected to ambient carbon monoxide; STB, serum total bilirubin.

The ROC curves are shown in Figure 1. Regarding the two parameters for predicting a long phototherapy duration, at a specificity of 80%, the sensitivity for STB alone, ETCOc alone, and the combination of both were 31.3%, 68.8%, and 68.8%, respectively. At a specificity of 50%, the sensitivities increased to 68.8%, 81.3%, and 81.3%, respectively. The AUC of STB alone, ETCOc alone, and the combination of both at admission were 0.577, 0.765, and 0.777, respectively(Table 3). Positive and negative predictive values (PPV and NPV) were also calculated. The cutoff value of ETCOc at admission was 2.4 ppm with a sensitivity of 62.5% and specificity of 88.5%, yielding a PPV of 50% and an NPV of 92.7%. With these levels of sensitivity and specificity, ETCOc was a reliable predictor of prolonged phototherapy duration (Table 4).

**Table 3:** AUC of ETCOc, STB, and the combination of both.

	AUC	*P*-value	95% CI
admSTB	0.577	0.33	0.424–0.73
admETCOc	0.765	0.001	0.624–0.906
admSTB + admETCOc	0.777	0.001	0.643–912

AUC, area under the curve; ETCOc, end-tidal carbon monoxide corrected to ambient carbon monoxide; STB, serum total bilirubin; adm, at admission.

**Table 4:** Sensitivity, specificity, PPV, and NPV for ETCOc greater than 2.4 ppm.

ETCOc >2.4 ppm	Phototherapy duration >72		
	Positive (16)	Negative (87)	
Positive (20)	True positive (TP) (10)	False positive (FP) (10)	PPV 10/20 = 50%
Negative (83)	False negative (FN) (6)	True negative (TN) (77)	NPV 77/83 = 92.7%
	Sensitivity 10/16 = 62.5%	Specificity 77/87 = 88.5%	

Sensitivity = TP/TP + FN, the probability of the test finding the disease among those with the disease or the proportion of those with the disease who have a positive test result. Specificity = TN/TN + FP, the probability of the test finding no disease among those who do not have the disease or the proportion of those free of the disease who have a negative test result. PPV = TP/(TP + FP), the percentage of people with a positive test result who actually had a phototherapy duration >72 h. NPV = TN/(FN + TN), the percentage of people with a negative test result with phototherapy duration ≤72 h.

Abbreviations: PPV, positive predictive value; NPV, negative predictive value; ETCOc, end-tidal carbon monoxide corrected to ambient carbon monoxide.

### Case reports

**Figure 2:**
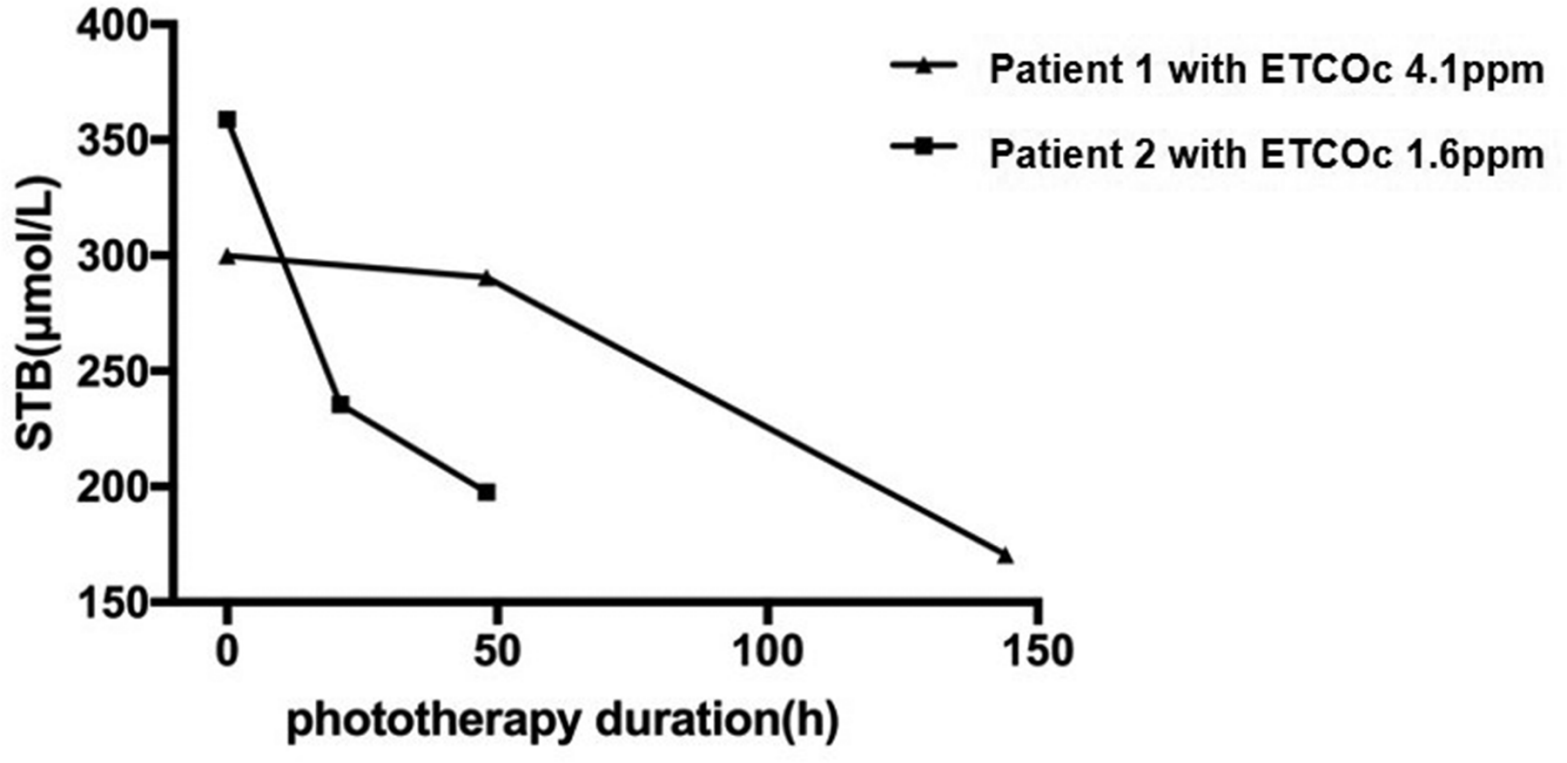
Dynamic changes in STB (serum total bilirubin)in two patients. Patient 1 was a full-term neonate (GA, 41 weeks and 2 days) with a BW of 3450 g. His ETCOc level was 4.1 ppm. After about 48 h of phototherapy, the STB decreased to 290.5 µmol/L. After 144 h of phototherapy, the STB was 170.6 µmol/L. Patient 2 was born at a GA of 39 weeks and 5 days with a BW of 3450 g. He was admitted with an STB of 358.8 µmol/L and an ETCOc of 1.6 ppm. After 21 h of phototherapy, the STB decreased to 235.5 µmol/L. After 48 h of phototherapy, the STB decreased to 197.5 µmol/L. STB, serum total bilirubin; GA, gestational age; BW, birth weight; ETCOc, end-tidal carbon monoxide corrected to ambient carbon monoxide.

Two male infants were admitted with significant nonhemolytic hyperbilirubinemia on the third day of life. Patient 1 was a full-term infant (GA, 41 weeks and 2 days) who was delivered *via* spontaneous vaginal delivery with a BW of 3450 g. His ETCOc was 4.1 ppm and the STB was 299.8 µmol/L. After approximately 48 h of phototherapy, the STB decreased to 290.5 µmol/L (the rate of STB decrease was 0.19 µmol/L/h). After 144 h of phototherapy, STB reduced to 170.6 µmol/L. Patient 2 was born at a GA of 39 weeks and 5 days with a BW of 3450 g. He was admitted with an STB of 358.8 µmol/L and ETCOc of 1.6 ppm. After 21 h of phototherapy, the STB decreased to 235.5 µmol/L (rate of STB decrease was 5.87 µmol/L/h). After 48 h of phototherapy, the STB level decreased to 197.5 µmol/L. No hemolytic disease or cephalohematoma was observed in both cases. Figure 2 shows the dynamic changes in the STB of both patients. Both neonates had similar GAs and initial STBs, but Patient 1 had a significantly higher ETCO than did Patient 2. Hence, the decrease in STB in Patient 1 was remarkably slower than that in Patient 2. This implies a longer phototherapy course (144 h vs. 48 h). The therapeutic procedures in these cases adequately demonstrated that the initial ETCOc, rather than the STB,predicts the duration of phototherapy.

## Discussion

ETCOc (4, 5, 13, 17, 18) is non-invasive and convenient to conduct and can be widely used in clinical practice. Its safety and feasibility are the premise of this trial. In our study, ETCOc was used in 103 enrolled infants without any adverse effects, and the whole process of administering non-invasive ETCOc tests was completed in only a few minutes and did not interfere with any treatment received by the patients.

As early as 20 years ago, ETCOc was used in clinical practice. Balaraman V et al. (19) reported that ETCOc levels decreased during the first several days, from 1.6 + ppm to 0.8 ppm, reflecting a physiologic breakdown of red blood cells. Maisels and Kring (20) indicated that there were sustained or increased ETCOc levels in infants with jaundice during the first 4 days, suggesting that increased heme catabolism is an important risk factor for the development of hyperbilirubinemia. Higher ETCOc values ≥1.8 ppm are also suggestive of hemolysis and are associated with significant hyperbilirubinemia (17). Our study indicated significant hyperbilirubinemia in infants admitted at 3–7 days with a mean ETCOc value of 1.7 ppm. This observation is consistent with that in earlier studies.

Most current studies use early ETCOc values to predict the occurrence of subsequent hyperbilirubinemia (3, 12, 20) or to evaluate the relationship between ETCOc and hemolytic diseases (4, 5, 17). A 2021 study (21) tried to explore the relationship between ETCOc levels and the duration of phototherapy in infants with hyperbilirubinemia, including the time of phototherapy initiation as well as the number and duration of phototherapy courses; and the study mainly focused on the observation of normal newborns and only a small part of neonates who needed phototherapy with lower STB (mean 239.4 µmol/L). Our study included infants with significant hyperbilirubinemia and STB that required phototherapy for the given age and risk category (mean 327.0 µmol/L) and admission to the neonatal department for intense phototherapy. We clarified the relationship between ETCOc values and the duration of phototherapy.

We compared two patients with similar GA, BW, and age at admission, and found that patients with higher ETCOc on admission required a longer duration of phototherapy. This finding is consistent with our hypothesis. The reason that initial ETCOc but not STB measurements were a predictor of treatment duration is unclear. It is also unclear whether this conclusion necessarily extends to patients with mild hyperbilirubinemia. On the basis of our observations, we assumed that some cases of hyperbilirubinemia with mildly elevated ETCOc levels were due to insufficient bilirubin elimination. However, the decrease in bilirubin elimination might be transient, and STB could decrease in a relatively short time after phototherapy. In contrast, jaundice caused by increased bilirubin production has a remarkable increase in ETCOc, which cannot be compensated for by the restoration of bilirubin elimination capacity. Therefore, STB in the latter patients decreases slowly and requires a longer duration. Unfortunately, no technology is currently available to dynamically monitor the ability of a neonate to eliminate hepatic bilirubin. Given that ETCOc is a relatively non-invasive test that does not require blood sampling, it should likely be used in practice more widely, and it is particularly recommended for all patients receiving phototherapy prior to treatment.

In a study by Butani on the use of ETCOc values to predict hemolytic diseases (6), it was noted that the combination of STB, bilirubin elimination, and ETCOc approximately classifies all newborns into four categories, which may alert clinicians to certain neonates at risk of hemolysis or metabolic diseases. The fourth group of patients (characterized by high STB, ETCOc ≥ 2.0, and insufficient bilirubin elimination) had the highest risk of severe hyperbilirubinemia and bilirubin-induced neurological damage; however, the relationship between ETCOc and subsequent treatment was not further elaborated in this study. Our study focused on the significance of ETCOc values in neonates with hyperbilirubinemia (the latter two categories proposed by Butani).Moreover, in subsequent studies, we aim to monitor changes in STB and to extrapolate the STB-derived clearance rate, which can help neonatologists to fully understand internal bilirubin production and elimination in these infants.

**Figure 3:**
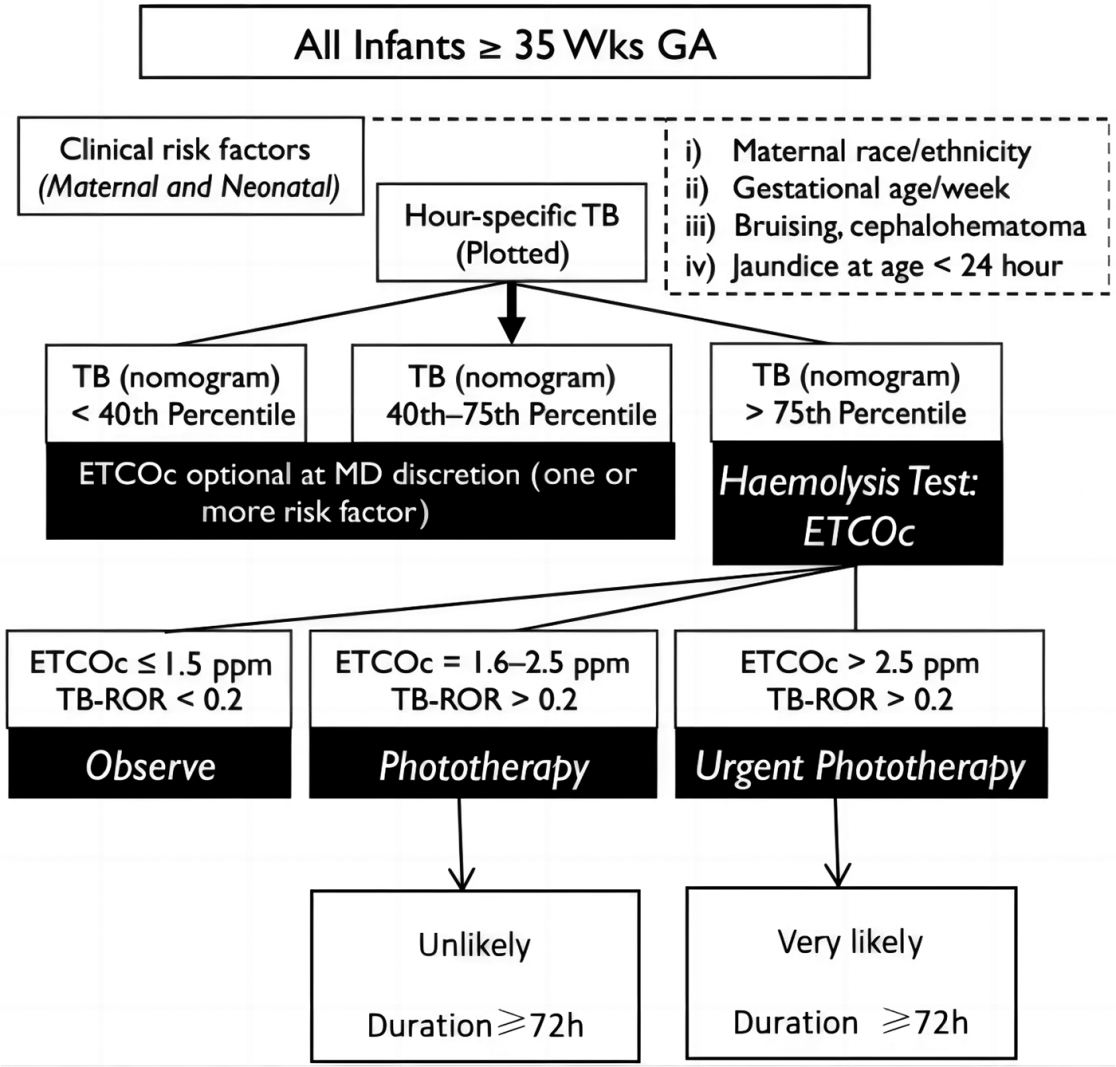
Clinical interpretative algorithm (supplementary version).TB-ROR, total serum/plasma bilirubin rate of rise (mg/dl/h; GA, gestational age; TB, total bilirubin; ETCOc, end-tidal carbon monoxide corrected to ambient carbon monoxide).

Our results showed that prolonged phototherapy was significantly associated with a high initial ETCO value. ETCOc (≥2.4 ppm) on admission was a good predictor, with a sensitivity of 62.5% and specificity of 88.5%. Based on these findings, clinicians can inform parents of newborns with a high ETCOc, especially cases with ETCOc >2.4 ppm, to anticipate the possible need for a longer duration of phototherapy and stay in the neonatology department. Butani et al. (4) described a clinical interpretative algorithm to summarize the treatment flow path of icteric newborns with a GA of >35 weeks. Our findings might supplement this, regarding the duration of phototherapy. As shown in Figure 3, patients with ETCO > 2.5 ppm and TB-ROR > 0.2 are very likely receive phototherapy for >72 h.

This study had several limitations. Possible selection bias existed due to short trial duration and limited sample size. The present study focused on children between 3 and 7 days of age with the goal of reducing the impact of the physiological processes of red cell destruction that is prevalent in the early neonatal period. Further studies could incorporate more suitable neonates to understand the physiological and pathological processes of bilirubin production.

## Conclusions

In conclusion, the results of our study verified the effective role of ETCOc in the prediction of phototherapy duration in infants with hyperbilirubinemia, and indicated that the assessment of disease severity using ETCOc values is extremely meaningful, not only for physicians to make treatment plans but also to improve the adherence of the children's families to these plans.

## Data Availability

The raw data supporting the conclusions of this article will be made available by the authors, without undue reservation.
